# Role of *Mycobacterium tuberculosis pknD *in the Pathogenesis of central nervous system tuberculosis

**DOI:** 10.1186/1471-2180-12-7

**Published:** 2012-01-13

**Authors:** Nicholas A Be, William R Bishai, Sanjay K Jain

**Affiliations:** 1 Center for Tuberculosis Research, Johns Hopkins University School of Medicine, 1550 Orleans Street, Baltimore, Maryland, 21287, USA; 2 Department of Pediatrics, Johns Hopkins University School of Medicine, 1550 Orleans Street, Baltimore, Maryland, 21287, USA; 3 Center for Infection and Inflammation Imaging Research, Johns Hopkins University School of Medicine, 1550 Orleans Street, Baltimore, Maryland, 21287, USA

## Abstract

**Background:**

Central nervous system disease is the most serious form of tuberculosis, and is associated with high mortality and severe neurological sequelae. Though recent clinical reports suggest an association of distinct *Mycobacterium tuberculosis *strains with central nervous system disease, the microbial virulence factors required have not been described previously.

**Results:**

We screened 398 unique *M. tuberculosis *mutants in guinea pigs to identify genes required for central nervous system tuberculosis. We found *M. tuberculosis pknD *(*Rv0931c*) to be required for central nervous system disease. These findings were central nervous system tissue-specific and were not observed in lung tissues. We demonstrated that *pknD *is required for invasion of brain endothelia (primary components of the blood-brain barrier protecting the central nervous system), but not macrophages, lung epithelia, or other endothelia. *M. tuberculosis pknD *encodes a "eukaryotic-like" serine-threonine protein kinase, with a predicted intracellular kinase and an extracellular (sensor) domain. Using confocal microscopy and flow cytometry we demonstrated that the *M. tuberculosis *PknD sensor is sufficient to trigger invasion of brain endothelia, a process which was neutralized by specific antiserum.

**Conclusions:**

Our findings demonstrate a novel *in vivo *role for *M. tuberculosis pknD *and represent an important mechanism for bacterial invasion and virulence in central nervous system tuberculosis, a devastating and understudied disease primarily affecting young children.

## Background

Tuberculosis (TB) of the central nervous system (CNS) is a devastating and often fatal disease, primarily affecting young children. Even when treatment is administered in a timely manner, mortality is extraordinarily high, with surviving patients often experiencing severe neurological sequelae. CNS TB comprises approximately 1% of TB disease worldwide, disproportionately affecting children in developing nations [1]. Coinfection with human immunodeficiency virus increases the likelihood of CNS TB [2,3], and the emergence of drug resistant strains further complicates CNS TB due to limited permeability at the blood-brain barrier (BBB) of several second-line TB drugs. Delays in treatment due to drug-susceptibility testing further reduce the efficacy of available patient care [4].

The CNS is protected from the systemic circulation by the BBB, composed principally of specialized and tightly apposed brain microvascular endothelia (BMEC), supported by astrocyte processes [5,6]. According to the widely accepted hypothesis by Rich *et al *(1933), lesions (Rich foci) develop around bacteria seeded in the brain parenchyma and meninges during the initial hematogenous dissemination. Subsequent rupture of these foci results in the release of bacteria directly into the CSF, causing extensive inflammation and meningitis [7]. The onset of meningitis is most commonly observed in young children (between the ages of 0 and 4), and is also associated with HIV co-infection or recent corticosteroid use [8]. In addition to host risk factors, recent clinical reports have indicated the association of distinct *Mycobacterium tuberculosis *strains with CNS disease [9-12], and microbial factors which promote CNS disease have been identified in numerous other neuroinvasive pathogens [13]. While it is clear that *M. tuberculosis *invade the CNS and that microbial factors may be required for CNS disease, the identity of such virulence determinants remains elusive.

We previously described a murine model of CNS TB utilizing hematogenous dissemination to the CNS, and identified *M. tuberculosis *genes required for CNS disease [14]. We developed a similar model of CNS TB in the guinea pig, which, unlike mice, develop well-defined, necrotic granulomas in response to *M. tuberculosis *infection [15], and were also utilized by Rich *et al *for their seminal work on CNS TB [7]. By screening and characterizing several hundred *M. tuberculosis *transposon (Tn) mutants, we identified *M. tuberculosis pknD *as a key microbial factor required for CNS disease.

## Results

### M. tuberculosis *genes required for CNS disease*

Guinea pigs were infected by intravenous injection of 1 × 10^6 ^*M. tuberculosis*. Animals become moribund and succumb to pulmonary and disseminated disease 24-28 days after such an infection, thus 21 days was chosen as the end-point for our mutant screens. Whole brain CFU were reliably > 1 × 10^4 ^CFU at day 21.

398 genotypically-defined *M. tuberculosis *Tn disruption mutants, each with a disruption in a single gene were screened (Additional file 1). The mutant output pool (bacilli harvested from lungs and brains at day 21) was compared to input pool (bacilli harvested from blood on the day of infection). Mutants attenuated in the CNS were also tested for their survival phenotype in the lung tissue. Of the 398 mutants analyzed, 14 were found to exhibit CNS-specific attenuation (> 16 fold). No corresponding defects were observed for these mutants in lung tissue (< 4 fold attenuation) (Table 1). Similar results were obtained when attenuation in the brain was compared with lung tissues (instead of blood on the day of infection). One of the 14 mutants identified in the screen, *M. tuberculosis pknD *(*Rv0931c*), was highly attenuated in the guinea pig brain, and was also identified to have a CNS-specific phenotype in our previously reported work utilizing the murine model [14]. Polar effects on the predicted operon partner *pstS2 *are not expected, as *pknD *is located downstream of *pstS2*. Additionally, nearby downstream genes are oriented in the opposite direction to *pknD*.

**Table 1:** *M. tuberculosis *genes found to be associated with CNS invasion/survival in the guinea pig

Gene MT #	Gene Rv #	Description	P value
MT0086	Rv0079	Conserved Hypothetical Protein	5.87E-04

MT0350	Rv0336	Conserved 13E12 Repeat Family Protein	1.99E-03

MT0752	Rv0727c	Possible Aldolase	5.07E-04

MT0779	Rv0755c	PPE Family Protein	1.25E-04

MT0958	Rv0931c	Ser-Thr Protein Kinase (PknD)	1.65E-03

MT1311	Rv1273c	Probable Drug-Transport ABC Transporter	2.71E-04

MT1711	Rv1673c	Conserved Hypothetical Protein	ND

MT1965	Rv1914c	Conserved Hypothetical Protein	ND

MT1982	Rv1932	Probable Thiol Peroxidase Tpx	4.47E-05

MT2456	Rv2387	Conserved Hypothetical Protein	2.53E-04

MT3178	Rv3094c	Conserved Hypothetical Protein	ND

MT3247	Rv3159c	PPE Family Protein	5.87E-05

MT3321	Rv3224	Iron-Regulated Dehydrogenase/Reductase	2.77E-03

MT3461	Rv3353c	Conserved Hypothetical Protein	9.12E-03

Mutants for the above genes were found to be significantly attenuated in the guinea pig CNS, but not lungs, 21 days after intravenous infection. Selected mutants were attenuated greater than 16-fold in the CNS and less than 4-fold in the lung tissue (P < 0.05). Mutants annotated as "ND" were not detected in the brain in quantities detectable by PCR, and were therefore likely highly attenuated in CNS tissue.

**Figure 1:**
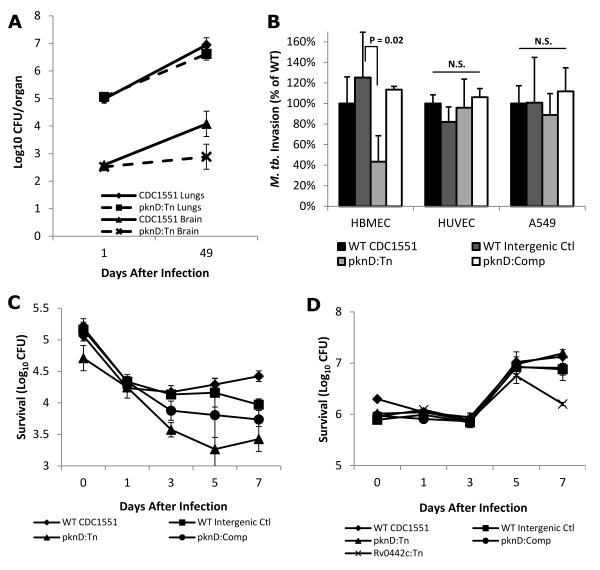
**Invasion and survival of *M. tuberculosis pknD *mutant in host-derived cells**. **A**. BALB/c mice were infected with *M. tuberculosis *CDC1551 or *pknD *mutant, and sacrificed at days 1 and 49 after infection. The mutant for *M. tuberculosis pknD *was significantly attenuated (P = 0.004) in mouse brain, but not lung tissue, 49 days after infection. No defect was observed in the lungs at either time point. Bacterial burden is represented as log_10 _CFU/organ for all animal experiments. **B**. Invasion of host-cell monolayers by wild-type CDC1551, wild-type intergenic transposon control, *pknD *transposon mutant (pknD:Tn), and *pknD *genetic complement (pknD:Comp) was examined and normalized to the wild-type control. Invasion assays were performed in brain microvascular endothelial cells (HBMEC), epithelial A549 cells, and umbilical vein endothelia (HUVEC). No difference in invasion was observed in A549 cells (P = 0.31) or HUVEC (P = 0.41). A significant reduction in invasive capacity, however, was observed in the CNS-derived HBMEC (P = 0.02). This defect was restored by genetic complementation with the native *pknD/pstS2 *operon. N.S. = not significantly different. **C**. Intracellular survival of each of the above *M. tuberculosis *strains was examined in HBMEC at days 1, 3, 5, and 7 after infection. The pknD:Tn mutant demonstrated an invasion and intracellular survival defect in HBMEC relative to wild-type over the course of the seven day infection. **D**. Survival was also examined by infection of activated J774 macrophages. No corresponding survival defect for the pknD:Tn mutant was observed in these cells during the seven day infection. A mutant for the gene *Rv0442c*, known to be attenuated in the macrophage model, is included as a control. All CFU counts are represented as mean ± standard deviation.

To verify our results from the pooled infections, we tested the *M. tuberculosis pknD *mutant individually. Mice were intravenously infected with *M. tuberculosis *wild-type or *pknD *mutant strains and sacrificed at days 1 and 49 following infection. Equal numbers of the *M. tuberculosis *wild-type and *pknD *mutant strains were implanted at day 1 in the brain (2.58 ± 0.07 and 2.52 ± 0.07 log_10 _CFU; P = 0.61) and lungs (4.98 ± 0.14 and 5.06 ± 0.15 log_10 _CFU; P = 0.50) respectively (Figure 1A). Note that even though a modest invasion defect is expected for the *pknD *mutant, the *in vivo *models are not powered to reliably observe these modest differences at day 1, which, however, are amplified by day 49. The *M. tuberculosis pknD *mutant was significantly attenuated for survival in the brain (18.7 fold), compared to the wild-type strain (P = 0.004), but not in the lung tissue (Figure 1A). Taken together with our observations during pooled infection in both mice and guinea pigs, these data indicate a CNS-associated defect for the *M. tuberculosis pknD *mutant.

### M. tuberculosis pknD *is necessary for invasion of CNS-derived endothelia*

To determine whether the observed phenotype was due to a specific interaction with host cells likely to encounter *M. tuberculosis *in CNS or lung tissues, invasion assays were performed in activated J774 macrophages and non-professional phagocytic cells [CNS-derived BMEC (HBMEC), A549 alveolar basal epithelial cells, and umbilical vein endothelia (HUVEC)]. HUVEC and A549 were chosen as they represent the most commonly used endothelial and pulmonary epithelial cells, respectively, employed for pathogen studies. Infections were performed with *M. tuberculosis *wild-type, *pknD *mutant, or a strain which was complemented with the *pknD/pstS2 *operon. Strain CQ0688, an intergenic *M. tuberculosis *Tn mutant, was used as a negative control, while *M. tuberculosis Rv0442c *mutant, known to be attenuated in macrophages [16], was used as a positive control for macrophage experiments. The *pknD *mutant demonstrated an invasion defect in HBMEC after 90 minutes of infection (P = 0.02), a defect restored by complementation (Figure 1B). These results were confirmed in three independent experiments. Invasion of A549 or HUVEC by the *pknD *mutant was not significantly lower than that of wild-type (Figure 1B).

Since macrophages are the key host cells that interact with *M. tuberculosis *in the lungs, bacterial survival assays were also performed to assess the role of *pknD *in activated J774 macrophages. Host cells were lysed and bacteria cultured at days 0, 1, 3, 5, and 7 following infection. Bacterial counts for the *pknD *mutant remained below that of wild type bacteria in HBMEC at days 3 (P = 0.008), 5 (P = 0.03), and 7 (P = 0.003) during the course of the infection (Figure 1C). When accounting for the reduced invasion at day 0, an intracellular survival defect was still observed at days 5 (P = 0.03) and 7 (P = 0.03). No corresponding defect was observed for the *pknD *mutant at any time point in macrophages (Figure 1D). These data indicate that the CNS-associated defect of the *pknD *mutant may be due to defective invasion and survival in brain endothelia.

### The PknD extracellular domain is sufficient to trigger adhesion and invasion of brain endothelia

**Figure 2:**
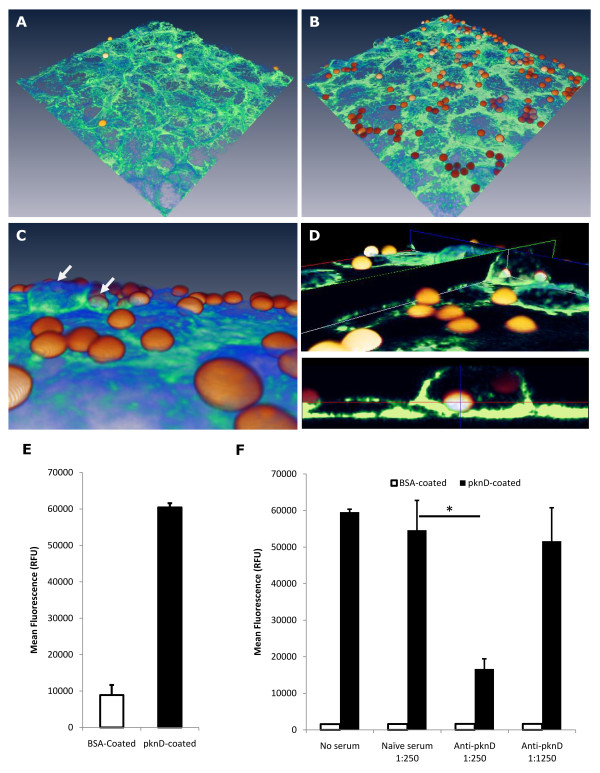
***M. tuberculosis *PknD is sufficient to trigger adhesion to HBMEC**. **A and B**. Fluorescent microspheres were coated with either PknD sensor or BSA, inoculated into HBMEC, washed, and stained for actin. Confocal microscopy demonstrated that PknD sensor-coated microspheres (panel B) adhere to brain endothelia to a greater degree than those coated with BSA (panel A). **C**. Confocal images were assembled into a 3D reconstruction and examined under higher magnification. PknD sensor-coated microspheres appear to be largely enveloped by actin processes (arrows) indicating that PknD-induced uptake by host cells may be an active process. **D**. When confocal images are examined in multiple planes, it is clear that a number of microspheres exist intracellularly. **E**. Wells containing endothelial cells with microspheres were analyzed for fluorescence. Quantification of fluorescence demonstrated a significant increase in the adherence of PknD-coated microspheres to the monolayer (P = 0.0002). **F**. Microspheres were pre-incubated with either custom anti-PknD serum or naïve serum. Incubation with anti-PknD serum (1:250 dilution) significantly reduced adherence of PknD (P = 0.0007) but not BSA-coated microspheres (P = 0.6). Moreover, no reduction in adherence was noted for PknD or BSA-coated microspheres when incubated with naïve antiserum (BSA: P = 0.4; PknD: P = 0.1; ANOVA single factor). Fluorescence readings are presented as mean ± standard deviation. *Statistically significant difference.

In order to determine whether the presence of PknD protein is sufficient for invasion, fluorescent microspheres were coated with either recombinant PknD sensor or bovine serum albumin (BSA). Host cell actin cytoskeleton was stained with Alexafluor 488-Phalloidin. Coated microspheres were incubated with brain endothelia (HBMEC) for 90 minutes, followed by extensive washing. Confocal microscopy demonstrated that higher numbers of PknD-coated microspheres adhered to HBMEC than in the case of BSA-coated control microspheres (Figure 2A-B). Additionally, the PknD-coated microspheres were largely embedded within indentations of actin and near sites of actin protrusion (Figure 2C). Several microspheres were visually confirmed to be intracellular after the inoculation (Figure 2D). A significant increase in fluorescence was observed in wells containing PknD-coated microspheres relative to those containing their BSA-coated counterparts (P = 0.0002) (Figure 2E). Adherence of PknD-coated microspheres (but not BSA-coated microspheres) to HBMEC was significantly reduced by pre-incubation with anti-PknD serum, when compared to incubation with naïve antiserum (P = 0.005) (Figure 2F).

**Figure 3:**
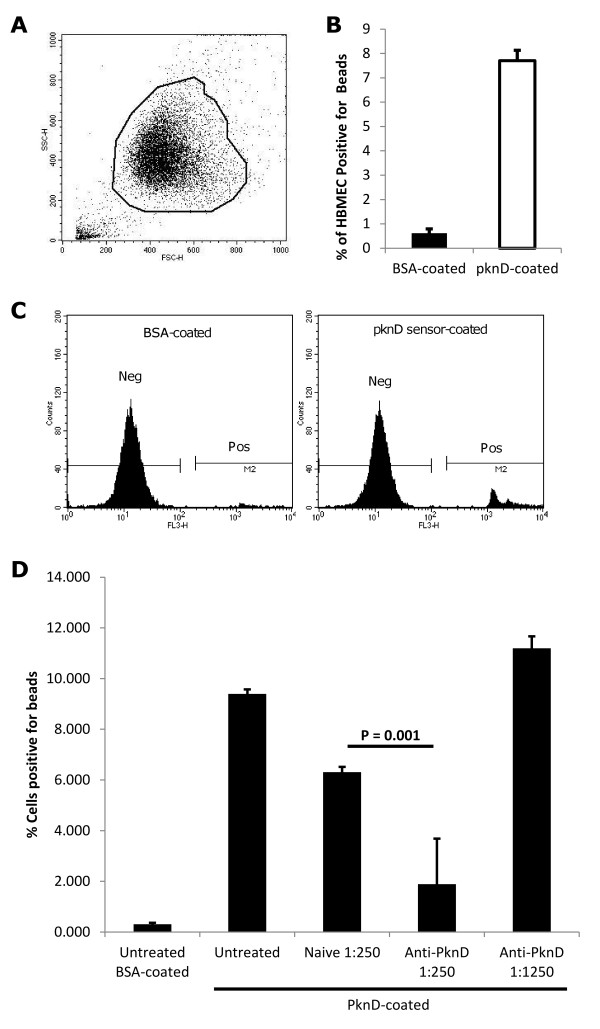
***M. tuberculosis *PknD triggers invasion of the brain endothelium**. **A**. Brain endothelia were inoculated with either PknD sensor- or BSA-coated fluorescent microspheres, washed, and disrupted by trypsinization. Endothelia were subjected to flow cytometry and gated to remove extracellular microspheres not associated with whole cells. **B**. Flow cytometry analysis demonstrated that significantly more endothelial cells were positive for fluorescence when incubated with PknD sensor-coated microspheres compared to BSA-coated microspheres (7.7% vs. 0.6%; P = 0.0003). Cell counts are presented as mean ± standard deviation. **C**. Histograms show that discrete fluorescent-positive populations are evident in the cells inoculated with PknD sensor-coated microspheres, indicating that cell populations took up multiple quantities of microspheres. **D**. Microspheres were again pre-incubated with either custom anti-PknD serum or naïve serum, followed by inoculation onto endothelial cells. Pre-incubation with anti-PknD (1:250) significantly reduced the population of cells which were positive for fluorescent microspheres, compared to naïve serum, as is indicated in the figure by a horizontal bar (P = 0.001). Pre-incubation with anti-PknD (1:1250) had no effect on internalization, when compared to untreated cells (P = 0.07).

In order to determine whether microspheres were invading and present intracellularly, the above incubations were repeated, and cells analyzed by flow cytometry. We observed that, in samples incubated with PknD-coated microspheres, 7.7 ± 0.4% of HBMEC contained fluorescent spheres, while only 0.6 ± 0.2% of cells incubated with BSA-coated microspheres were positive for fluorescence (Figure 3A-C). Microspheres were again incubated with anti-PknD serum, and internalization by HBMEC was significantly reduced when compared to incubation with naïve serum (P = 0.001) (Figure 3D). Together, these data indicate that *M. tuberculosis *PknD is sufficient to trigger uptake by brain endothelia.

### M. tuberculosis pknD *mutant exhibits reduced adherence to a component of the host ECM*

**Figure 4:**
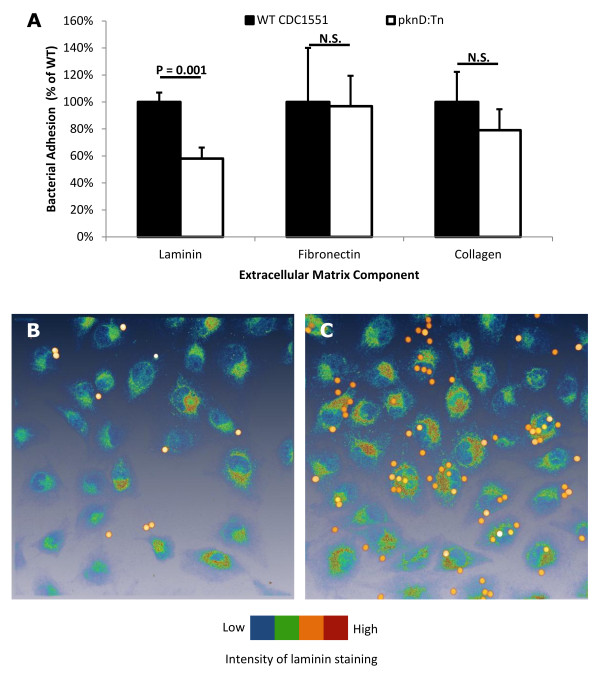
***M. tuberculosis *PknD sensor domain interacts with host laminin**. **A**. *M. tuberculosis *WT and *pknD *mutant were incubated in wells coated with components of the extracellular matrix (laminin, fibronectin, and collagen). The *pknD *mutant demonstrated a 2-fold reduction in adhesion to the laminin matrix (P = 0.001), while not exhibiting significantly reduced adhesion to fibronectin or collagen. CFU counts are represented as mean ± standard deviation. N.S. = not significantly different. **B and C**. Coated microspheres were incubated with HBMEC, followed by immunostaining for laminin. Microspheres coated with PknD sensor (panel C) associated with the periphery of laminin staining more than those coated with BSA (panel B), which were evenly distributed throughout the field of view.

Since *M. tuberculosis *PknD sensor is homologous to proteins that bind to the host ECM, we measured the adherence of *M. tuberculosis pknD *mutant to major components of the ECM using laminin, collagen, and fibronectin matrices generated *in vitro*. The *M. tuberculosis pknD *mutant demonstrated a reduction in association with the *in vitro *laminin matrix (P = 0.001), but not to collagen or fibronectin matrices (Figure 4A). Endothelia secrete laminin to generate a matrix for adhesion and maintenance of cell structure. To determine whether PknD protein associates with laminin secreted by brain endothelia, PknD-coated microspheres were incubated with HBMEC and stained for host laminin. It was observed that, relative to BSA-coated microspheres, PknD-coated microspheres were more likely to localize with the laminin-stained HBMEC (Figure 4B-C).

### *Invasion of brain endothelial cells by *M. tuberculosis *is reduced by *anti-PknD serum

**Figure 5:**
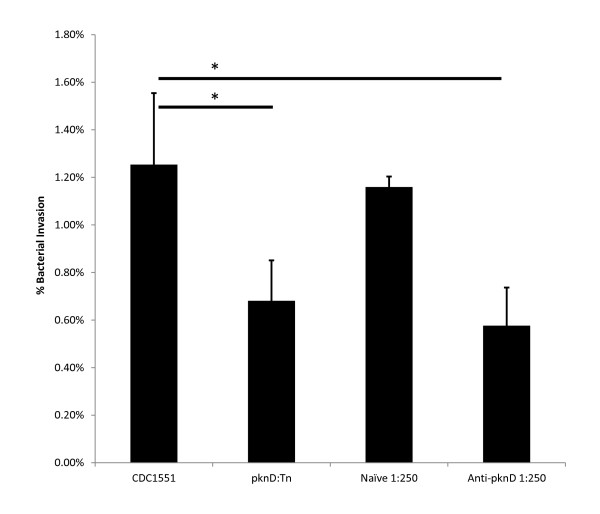
**Invasion of brain endothelia by *M. tuberculosis *is reduced by anti-PknD serum**. *M. tuberculosis *CDC1551 were pre-incubated with naïve or custom anti-PknD serum, washed, and used to infect brain endothelial cells. Following 90 minutes of infection, cells were lysed and CFU enumerated. It was observed that incubation with anti-PknD serum, but not naïve serum, significantly reduced the number of bacilli able to successfully invade HBMEC (P = 0.01). *Statistically significant difference.

Following observations that anti-PknD serum reduces adhesion and invasion of PknD-coated microspheres, similar experiments were performed using live *M. tuberculosis. M. tuberculosis *exposed to PknD-specific antibodies at a dilution of 1:250 were significantly attenuated in their ability to invade the brain endothelium relative to those bacteria incubated with naïve serum (P = 0.004) (Figure 5).

## Discussion

Recent clinical studies have observed the association of *M. tuberculosis *strains with CNS disease [9-12], and suggest that *M. tuberculosis *may possess virulence factors which promote CNS involvement. *M. leprae *ML-LBP21, for instance (a major surface protein), has been shown to be involved in Schwann cell invasion via laminin-2 [17], while *M. tuberculosis *malate synthase has been shown to bind ECM associated with A549 cells [18]. Additionally, the heparin-binding hemagglutinin of *M. tuberculosis *has been shown to be required for extra-pulmonary dissemination [19].

We utilized both the guinea pig and mouse models of hematogenous dissemination to the CNS in this study. In previous experiments with single strain infections, we have regularly observed a high degree of bacillary invasion of the guinea pig CNS. When performing an intravenous infection, we can reliably reproduce conditions where greater than 50,000 bacilli are present in the brain over a 3 week infection. Whole brain CFU in the mouse after an intravenous infection are lower than in the guinea pig [14]. This is important during our pooled infections when 100 mutants are simultaneously injected as we need an adequate total bacillary burden to provide sufficent numbers of each individual mutant. A burden of 50,000, for instance, would yield approximately 500 bacilli for each mutant. If only 50 bacilli were present (as may be seen in the mouse model), we would likely not be able to draw definite conclusions. This was not a concern during single mutant infections, as only one strain was present. We therefore used the mouse, which is also a reliable model [14], and is more feasible for performing the single strain infections. An additional benefit of using multiple animal systems is the validation provided by replicating our findings in several *in vivo *models. As described above, the *M. tuberculosis pknD *mutant was found to be highly attenuated in both animal models.

Since the CNS is protected from the systemic circulation by the BBB, *M. tuberculosis *can initiate CNS TB by crossing the BBB as extracellular organisms or via infected monocytes or neutrophils. While the latter hypothesis seems attractive, such cellular traffic is severely restricted across the barrier before the onset of TB meningitis [20]. Moreover, patients with CNS TB and meningitis have extensive blood vessel involvement and significant endovasculitis with the intima (comprising brain endothelia) most severely affected [21]. Goldzieher *et al*. have further shown that *M. tuberculosis *can be found inside brain endothelia of patients with TB meningitis [22]. Seminal work by Rich *et al*, later confirmed by MacGregor and colleagues, demonstrated that free *M. tuberculosis *can invade the CNS [7,23]. More modern data utilizing CD18-/- leukocyte adhesion deficient mice suggest that free mycobacteria can traverse the BBB independent of leukocytes or macrophages [24]. These data emphasize the central role of brain endothelia in the pathogenesis of CNS TB and underscore the importance of our observation that the *pknD *mutant displayed defective invasion and reduced survival in brain endothelia. While endothelial cells are not professionally phagocytic, they are capable of mounting an antibacterial response through the release of antimicrobial peptides. Activation of endothelial barriers can also trigger bacterial killing via NO- or H_2_O_2_-dependent pathways [25,26]. It is possible that disruption of *pknD *disables a bacterial response pathway necessary for survival in these unique conditions, resulting in the reduced intracellular growth we observed during infection of brain endothelial cells.

Reduced invasion was not observed in other cells previously utilized to evaluate invasion and dissemination defects of *M. tuberculosis *mutants and clinical strains [19,27]. However, one of the limitations of the current study is that other CNS cell types such as microglia and astrocytes, which could play a role in mycobacterial infection and killing *in vivo*, were not evaluated.

*M. tuberculosis pknD *encodes a "eukaryotic-like" STPK, a family of bacterial signaling proteins. STPKs occur in numerous pathogenic bacteria, and *M. tuberculosis *encodes 11 putative STPKs (*pknA-L*). Good *et al *have demonstrated that the *M. tuberculosis *PknD sensor is composed of a highly symmetric six-bladed β-propeller forming a cup with a functional binding surface [28]. The β-propeller is a widespread motif found mostly in eukaryotes, although it was first described in influenza virus neuraminidase [29]. Takagi *et al *have shown that nidogen, a β-propeller-containing protein in humans which is homologous to the sensor domain of *M. tuberculosis *PknD, is required for binding to laminin [30]. Similarly, *Trypanosoma cruzi*, a protozoan pathogen that causes meningoencephalitis in humans, has a PknD homolog (Tc85-11), also possessing a β-propeller, that selectively binds to laminin [31]. In accordance with bioinformatics predictions, *M. tuberculosis *PknD has been identified as an integral membrane protein in several proteomics studies [32,33]. We therefore hypothesized that the PknD sensor domain could be assisting in bacterial adherence by association with host ECM components, and our *in vitro *data are consistent with this hypothesis. Moreover, since brain endothelia associate principally with laminin 1 and 2, not present in epithelia and endothelia elsewhere [13,34,35], we postulate that the observed CNS tropism of *pknD *may be due to its interaction with CNS-associated laminin isoforms.

Bacterial STPKs are candidates for sensing the environment and regulation of microbial metabolic states [36,37]. The *M. tuberculosis *PknD intracellular kinase has been previously demonstrated to associate with and phosphorylate intracellular targets including MmpL7 [38] and the putative anti-anti-sigma factor Rv0516c, regulating *sigF*-associated genes [39]. *M. tuberculosis sigF *is an alternative sigma factor implicated in stress response, stationary phase, dormancy, and late-stage disease *in vivo *[40,41]. Our previously published data demonstrate that *M. tuberculosis *significantly down-regulate transcription, protein synthesis, and energy metabolism very early after invasion by brain endothelia [42]. These data raise the possibility that interaction with the host CNS may mediate bacterial signaling. The two domain structure of PknD invites the hypothesis that an extracellular signal, possibly a host factor, may induce an intracellular cascade via activity of the kinase and regulation of *sigF*. An ortholog of *M. tuberculosis pknB *in *Bacillus subtilis *has been demonstrated to regulate bacterial dormancy by a similar mechanism [43,44]. The potential induction of *sigF*-mediated cellular activity via *pknD *could confer upon *M. tuberculosis *a survival advantage in unique conditions such as the brain endothelium.

*M. tuberculosis *are well known to adapt to a quiescent dormant state. However, the precise location of dormant bacilli during human latent TB infection remains elusive. Immune surveillance of foreign antigens is relatively limited in the CNS [20,45], and mycobacteria escape immune recognition following direct inoculation into the brain parenchyma [46]. We therefore postulate that the unique microenvironment in the CNS is advantageous for bacterial survival, and may provide a sanctuary to dormant *M. tuberculosis*. While this study examines and indicates a role for *M. tuberculosis pknD *in the initial stages of invasion and infection, the role of dormancy in CNS disease will be an active area of research for our future studies.

Given the above data, we hypothesize that interaction of PknD protein with a host extracellular factor, possibly laminin, facilitates adhesion of *M. tuberculosis *to the microvascular endothelium of the CNS. Other neurotropic pathogens have been shown to trigger host-mediated uptake and internalization of bacteria through cytoskeletal rearrangement, thus this represents a possible mechanism for future study [47,48]. Given the multi-domain structure of PknD, extracellular engagement of the sensor domain could transduce a signal to the intracellular kinase, triggering a bacterial state, possibly dormancy, which is more amenable to uptake and survival in the microenvironment of the CNS.

It should also be noted that the PknD sensor domain occurs only in pathogenic mycobacteria, and is present in all sequenced clinical strains. Polymorphisms in the *pknD *gene or its promoter could therefore account for variable CNS tropism of distinct lineages of *M. tuberculosis*. Studies evaluating polymorphisms in *M. tuberculosis *isolated from patients with CNS or pulmonary disease are currently underway and may shed light on the clinical relevance of *pknD *or other such genes potentially involved with promoting CNS TB. Finally, it is important to note that bacterial invasion of host cells could be neutralized by an antibody raised against the extracellular (sensor) domain of *M. tuberculosis *PknD. This is encouraging and suggests a potential role for PknD as a therapeutic target against CNS TB.

## Conclusions

We have identified several *M. tuberculosis *genes which play a role in CNS TB, and have discovered a novel biological function for *M. tuberculosis pknD *in CNS disease. Our findings were associated with CNS tissue, and were not observed in the lungs. We further found that *pknD *is required for invasion of cells lining the brain endothelium, and that the *M. tuberculosis *PknD sensor is sufficient to trigger invasion of brain endothelia. This process was neutralized by specific antiserum, which demonstrates promising therapeutic potential. These data present a unique and novel role for this serine-threonine protein kinase. Knowledge gained from further study of *pknD*, and other candidates identified in this study, may lead to the development of preventive strategies for CNS TB, a devastating and under-studied disease. Moreover, these studies may also shed light on extra-pulmonary reservoirs for dormant *M. tuberculosis*.

## Materials and methods

### M. tuberculosis *strains and media*

*M. tuberculosis *CDC1551 parent and mutant strains were grown at 37°C in 7H9 liquid broth (Difco) supplemented with oleic acid albumin dextrose catalase (BD), 0.5% glycerol, and 0.05% Tween 80. Mutants for pooled infections were grown in sealed 24 well plates. For colony counting, *M. tuberculosis *strains were plated onto Middlebrook 7H11 selective plates (BD). The *pknD *Tn mutant was complemented using the gene sequence corresponding to *pstS2 *and *pknD *(predicted operon), as well as 200 base pairs upstream of *pstS2 *to ensure inclusion of the full native *pknD *promoter. This sequence was cloned into plasmid pGS202, a single copy integrating plasmid, and transformed into the *pknD *Tn mutant.

### Pooled guinea pig infections

Mutant selection and pooled mutant infections were performed as described previously [14]. A pool complexity of 100 was used. Each pooled suspension was diluted to an OD_600 _of 0.1 in PBS and 200 uL injected intravenously into each of four Hartley guinea pigs (catheterized) corresponding to 1 × 10^6 ^bacilli per animal. Blood was obtained immediately following infection and cultured. Following 21 days of infection, guinea pigs were euthanized and perfused with saline. Blood, lungs, and whole brain were harvested, homogenized, and cultured. Bacterial colonies were pooled, and genomic DNA extracted.

### Quantitative PCR analyses

The frequency of individual mutants in each organ was assessed by qPCR (Bio-Rad) with mutant-specific primers spanning the transposon insertion junction. Samples were normalized to results from a set of primers amplifying a mutant-independent DNA sequence (sequence from *Rv0986*). Attenuation for each mutant in the CNS or lungs was expressed as the ratio of an individual mutant's quantity present in the input pool (blood sample immediately after infection) compared with the output pool (brain or lung sample 21 days after infection). All assays were performed at least in triplicate.

### Single mutant infection in the murine model

BALB/c mice were intravenously infected with 1 × 10^6 ^wild-type or *pknD *mutant strains, via the tail vein. Four animals were sacrificed for each group at days 1 and 49. Blood, lungs, and brain were extracted, homogenized, and cultured on 7H11 selective plates (BD) and colony forming units (CFU) obtained 4 weeks after sacrifice.

### Tissue culture and ex vivo infection

Primary human brain microvascular endothelial cells (HBMEC) were isolated, characterized and purified from the cerebral cortex of a 9 month old infant (IRB exempt) as previously described [49-51]. Cells were grown in RPMI 1640 media supplemented with 10% fetal bovine serum, 10% Nu Serum, L-glutamine, sodium pyruvate, MEM nonessential amino acids, and MEM vitamins as described previously [42]. J774 macrophages were grown in RPMI 1640 supplemented with 10% fetal bovine serum. Human umbilical vein endothelia (HUVEC) were grown in EBM-2 basal media containing EGM-2 MV SingleQuot supplements (Lonza). A549 cells were grown in DMEM supplemented with 10% FBS.

Infection of HBMEC with *M. tuberculosis *for invasion and intracellular survival assays was performed in triplicate at a multiplicity of infection (MOI) of 10:1 as described previously [14]. Macrophages were activated by addition of interferon-γ (IFN-γ) one day prior to infection and lipopolysaccharide (LPS) three hours prior to infection. The subsequent assay was then performed according to the same protocol used for HBMEC. Cells were inspected at each time point to ensure integrity of the monolayer, and extracellular bacteria were washed away prior to lysis of cells. Additionally, low levels of streptomycin were maintained in the media in order to preclude the possibility of extracellular growth.

For assays involving neutralization with antisera, bacteria were incubated with either naïve (pre-bleed) or anti-PknD serum for 60 minutes. Bacteria were subsequently washed in PBS and used for infections.

### Production and detection of PknD protein

The coding sequence for PknD amino acid residues 403-664 was cloned into pDEST17 (6 × N-terminal his-tag) using the Gateway cloning system (Invitrogen). Expression of PknD protein was induced using 0.1% L-arabinose at 37°C in BL21-AI *E. coli*. PknD protein was purified by SDS-PAGE and used to generate custom polyclonal antiserum in rabbits (Covance).

### Preparation and use of fluorescent microspheres

Protein was immobilized on 4 μm red fluorescent microspheres (Invitrogen). Recombinant PknD sensor domain protein or bovine serum albumin (BSA) were incubated with microspheres in phosphate buffered saline (PBS) at 25°C, using BSA as a blocking agent. Microspheres were added at a MOI of 1:1 and incubated for 90 minutes at 37°C and 5% CO_2_. Fluorescence readings (excitation 540 nm; emission 590 nm) were taken before and after washing. For flow cytometry, cells were trypsinized and processed on a FACSCalibur flow cytometer (BD). In the antiserum neutralization studies, microspheres were incubated with naïve serum (pre-bleed sera) or anti-pknD serum for 60 minutes, followed by washing and incubation with cells as described above. For confocal microscopy, cells were fixed in 4% formaldehyde and permeabilized. For actin staining, cells were incubated with Alexa Fluor-488 conjugated phalloidin (Invitrogen). For laminin immunostaining, cells were incubated with rabbit polyclonal antibody against murine laminin (Sigma-Aldrich) followed by FITC conjugated goat anti-rabbit IgG (Invitrogen).

### Adhesion to the extracellular matrix (ECM)

Laminin from EHS cells (laminin-1) (Sigma-Aldrich), fibronectin (Sigma-Aldrich), collagen (Invitrogen), or BSA (Sigma-Aldrich) were incubated at 100 ug/mL in 96-well ELISA plates (Greiner) at 25°C overnight in order to coat wells with a protein matrix. *M. tuberculosis *were incubated in these wells at 37°C for 90 minutes. Wells were washed, and the protein matrices disrupted by incubation with 0.05% trypsin. The suspensions were plated onto 7H11 plates.

### Statistical analysis

Statistical comparison between groups was performed using Student's *t *test and Microsoft Excel 2007. Multiple comparisons were performed using ANOVA single factor test and the Microsoft Excel 2007 Analysis Toolpak Add-in.

All protocols were approved by the Johns Hopkins University Biosafety and Animal Care and Use committees.

## Authors' contributions

NAB performed the experiments outlined within this study. SKJ and NAB conceptualized the studies' goals and designed experimental procedures. NAB, SKJ, and WRB analyzed and formatted the data. NAB and SKJ drafted the manuscript. SKJ and WRB provided funding and administrative support for the project. All authors read and approved the final manuscript.

## Supplementary Material

Additional file 1 **M. tuberculosis transposon disruption mutants screened for attenuation in the guinea pig model of central nervous system tuberculosis**. 398 transposon mutants were selected for pooled infection in the guinea pig model. Each mutant listed was screened for attenuation in the central nervous system. Click here for file
