# Supplementary material for: Role of Mycobacterium tuberculosis pknD in the Pathogenesis of central nervous system tuberculosis
Source: BMC Microbiol. 2012 Jan 13;12:7. doi: 10.1186/1471-2180-12-7 (PMC3322341; doi:10.1186/1471-2180-12-7)
Supplement: Additional file 1 — M. tuberculosis transposon disruption mutants screened for attenuation in the guinea pig model of central nervous system tuberculosis. 398 transposon mutants were selected for pooled infection in the guinea pig model. Each mutant listed was screened for attenuation in the central nervous system. [file 1471-2180-12-7-S1.PDF]

Additional file 1. *M. tuberculosis* transposon disruption mutants screened for attenuation in the guinea pig model of central nervous system tuberculosis.

Gene MT #	Gene Rv #	Description
MT0009	Rv0157A	
MT0015	Rv0012	PROBABLE CONSERVED MEMBRANE PROTEIN
MT0027	Rv0024	PUTATIVE SECRETED PROTEIN P60-RELATED PROTEIN
MT0074-1	Rv0068	PROBABLE OXIDOREDUCTASE
MT0074-2	Rv0068	PROBABLE OXIDOREDUCTASE
MT0076	Rv0070c	PROBABLE SERINE HYDROXYMETHYLTRANSFERASE GLYA2 (SERINE METHYLASE 2) (SHMT 2)
MT0085	Rv0078A	PROBABLE TRANSCRIPTIONAL REGULATORY PROTEIN
MT0086	Rv0079	HYPOTHETICAL PROTEIN
MT0091	Rv0084	POSSIBLE FORMATE HYDROGENLYASE HYCD (FHL)
MT0100	Rv0091	PROBABLE BIFUNCTIONAL MTA/SAH NUCLEOSIDASE
MT0113	Rv0104	CONSERVED HYPOTHETICAL PROTEIN
MT0119	Rv0110	PROBABLE CONSERVED INTEGRAL MEMBRANE PROTEIN
MT0120	Rv0111	POSSIBLE TRANSMEMBRANE ACYLTRANSFERASE
MT0134	Rv0126	TREHALOSE SYNTHASE TRES
MT0135	Rv0127	CONSERVED HYPOTHETICAL PROTEIN
MT0145	Rv0137c	PROBABLE PEPTIDE METHIONINE SULFOXIDE REDUCTASE MSRA
MT0156	Rv0148	PROBABLE SHORT-CHAIN TYPE DEHYDROGENASE/REDUCTASE
MT0160-1	Rv0151c	PE FAMILY PROTEIN
MT0160-2	Rv0151c	PE FAMILY PROTEIN
MT0168-1	Rv0159c	CONSERVED HYPOTHETICAL PROTEIN
MT0168-2	Rv0159c	PE FAMILY PROTEIN
MT0172	Rv0163	CONSERVED HYPOTHETICAL PROTEIN
MT0179	Rv0170	MCE-FAMILY PROTEIN MCE1B
MT0182	Rv0173	POSSIBLE MCE-FAMILY LIPOPROTEIN LPRK (MCE-FAMILY LIPOPROTEIN MCE1E)
MT0185	Rv0176	PROBABLE CONSERVED MCE ASSOCIATED TRANSMEMBRANE PROTEIN
MT0188	Rv0179c	POSSIBLE LIPOPROTEIN LPRO
MT0197	Rv0187	PROBABLE O-METHYLTRANSFERASE
MT0204	Rv0194	PROBABLE DRUGS-TRANSPORT TRANSMEMBRANE ATP-BINDING PROTEIN ABC TRANSPORTER
MT0213	Rv0203	POSSIBLE EXPORTED PROTEIN
MT0223	Rv0213c	POSSIBLE METHYLTRANSFERASE (METHYLASE)
MT0228	Rv0218	PROBABLE CONSERVED TRANSMEMBRANE PROTEIN
MT0230	Rv0220	PROBABLE ESTERASE LIPC
MT0232	Rv0222	PROBABLE ENOYL-COA HYDRATASE ECHA1
MT0254	Rv0240	CONSERVED HYPOTHETICAL PROTEIN
MT0264	Rv0250c	CONSERVED HYPOTHETICAL PROTEIN
MT0269	Rv0256c	PPE FAMILY PROTEIN
MT0282	Rv0269c	CONSERVED HYPOTHETICAL PROTEIN
MT0306	Rv0293c	CONSERVED HYPOTHETICAL PROTEIN

MT0324	Rv0311	CONSERVED HYPOTHETICAL
MT0335	Rv0320	POSSIBLE CONSERVED EXPORTED PROTEIN
MT0347		
MT0350	Rv0336	CONSERVED 13E12 REPEAT FAMILY PROTEIN
MT0357	Rv0342	ISONIAZID INDUCTIBLE GENE PROTEIN INIA
MT0399	Rv0386	PROBABLE TRANSCRIPTIONAL REGULATORY PROTEIN (PROBABLY LUXR/UHPA-FAMILY)
MT0404	Rv0393	CONSERVED 13E12 REPEAT FAMILY PROTEIN
MT0417-1	Rv0404	PROBABLE FATTY-ACID-COA LIGASE FADD30
MT0417-2	Rv0404	PROBABLE FATTY-ACID-COA LIGASE FADD30
MT0418	Rv0405	HYPOTHETICAL PROTEIN
MT0425	Rv0412c	POSSIBLE CONSERVED MEMBRANE PROTEIN
MT0469	Rv0453	PPE FAMILY PROTEIN
MT0472.1		
MT0474	Rv0458	PROBABLE ALDEHYDE DEHYDROGENASE
MT0482	Rv0466	CONSERVED HYPOTHETICAL PROTEIN
MT0487	Rv0470A	CONSERVED HYPOTHETICAL PROTEIN
MT0498	Rv0480c	POSSIBLE AMIDOHYDROLASE
MT0510	Rv0491	TWO COMPONENT SENSORY TRANSDUCTION PROTEIN REGX3
MT0523	Rv0502	CONSERVED HYPOTHETICAL PROTEIN
MT0534	Rv0513	POSSIBLE CONSERVED TRANSMEMBRANE PROTEIN
MT0539	Rv0518	POSSIBLE EXPORTED PROTEIN
MT0575	Rv0550c	HYPOTHETICAL PROTEIN
MT0589	Rv0563	PROBABLE PROTEASE TRANSMEMBRANE PROTEIN HEAT SHOCK PROTEIN HTPX
MT0593	Rv0567	PROBABLE METHYLTRANSFERASE/METHYLASE
MT0605	Rv0576	PROBABLE TRANSCRIPTIONAL REGULATORY PROTEIN (POSSIBLY ARSR-FAMILY)
MT0609	Rv0580c	CONSERVED HYPOTHETICAL PROTEIN
MT0611	Rv0583c	PROBABLE CONSERVED LIPOPROTEIN LPQN
MT0617	Rv0588	CONSERVED HYPOTHETICAL INTEGRAL MEMBRANE PROTEIN YRBE2B
MT0640	Rv0610c	HYPOTHETICAL PROTEIN
MT0642	Rv0612	PROBABLE AMIDOHYDROLASE AMIB1 (AMINOHYDROLASE)
MT0644	Rv0614	CONSERVED HYPOTHETICAL PROTEIN
MT0657	Rv0629c	PROBABLE EXONUCLEASE V (ALPHA CHAIN) RECD
MT0659	Rv0631c	PROBABLE EXONUCLEASE V (GAMMA CHAIN) RECC
MT0683	Rv0654	PROBABLE DIOXYGENASE
MT0688	Rv0659c	CONSERVED HYPOTHETICAL PROTEIN
MT0691	Rv0662c	CONSERVED HYPOTHETICAL PROTEIN
MT0700	Rv0671	POSSIBLE CONSERVED LIPOPROTEIN LPQP
MT0709	Rv0681	PROBABLE TRANSCRIPTIONAL REGULATORY PROTEIN (POSSIBLY TETR-FAMILY)
MT0715	Rv0687	PROBABLE SHORT-CHAIN TYPE DEHYDROGENASE/REDUCTASE
MT0723	Rv0696	PROBABLE MEMBRANE SUGAR TRANSFERASE
MT0749	Rv0724	POSSIBLE PROTEASE IV SPPA (ENDOPEPTIDASE IV) (SIGNAL PEPTIDE PEPTIDASE)

MT0752	Rv0727c	POSSIBLE L-FUCULOSE PHOSPHATE ALDOLASE FUCA (L-FUCULOSE-1-PHOSPHATE ALDOLASE)
MT0758	Rv0734	PROBABLE METHIONINE AMINOPEPTIDASE MAPA (MAP) (PEPTIDASE M) (METAP)
MT0778	Rv0754	PE-PGRS FAMILY PROTEIN
MT0779	Rv0755c	PPE FAMILY PROTEIN
MT0809	Rv0785	PROBABLE ENOYL-COA HYDRATASE ECHA1 (ENOYL HYDRASE)
MT0825	Rv0805	PHOSPHODIESTERASE
MT0837	Rv3117	PROBABLE THIOSULFATE SULFURTRANSFERASE CYSA3 (RHODANESE-LIKE PROTEIN)
MT0852	Rv0831c	CONSERVED HYPOTHETICAL PROTEIN
MT0858	Rv0837c	HYPOTHETICAL PROTEIN
MT0869	Rv0846c	PROBABLE OXIDASE
MT0879	Rv0856	CONSERVED HYPOTHETICAL PROTEIN
MT0890	Rv0867c	POSSIBLE RESUSCITATION-PROMOTING FACTOR RPFA
MT0894	Rv0872c	PE-PGRS FAMILY PROTEIN
MT0911	Rv0888	PROBABLE EXPORTED PROTEIN
MT0919	Rv0895	CONSERVED HYPOTHETICAL PROTEIN
MT0934	Rv0910	CONSERVED HYPOTHETICAL PROTEIN
MT0951-1	Rv0924c	DIVALENT CATION-TRANSPORT INTEGRAL MEMBRANE PROTEIN MNTH (BRAMP) (MRAMP)
MT0951-2	Rv0924c	DIVALENT CATION-TRANSPORT INTEGRAL MEMBRANE PROTEIN MNTH (BRAMP) (MRAMP)
MT0958	Rv0931c	SER-THR PROTEIN KINASE (PKND)
MT0967	Rv0940c	POSSIBLE OXIDOREDUCTASE
MT0975	Rv0948c	CONSERVED HYPOTHETICAL PROTEIN
MT0981	Rv0954	PROBABLE CONSERVED TRANSMEMBRANE PROTEIN
MT0992.1	Rv0964c	HYPOTHETICAL PROTEIN
MT0996	Rv0968	CONSERVED HYPOTHETICAL PROTEIN
MT1002	Rv0974c	PROBABLE ACETYL-/PROPIONYL-COA CARBOXYLASE (BETA SUBUNIT) ACCD2
MT1003.1	Rv0976c	CONSERVED HYPOTHETICAL PROTEIN
MT1004		
MT1006.1	Rv0978c	PE-PGRS FAMILY PROTEIN
MT1014	Rv0986	PROBABLE ADHESION COMPONENT TRANSPORT ATP-BINDING PROTEIN ABC TRANSPORTER
MT1025.3		
MT1030	Rv1001	PROBABLE ARGININE DEIMINASE ARCA (ADI) (AD) (ARGININE DIHYDROLASE)
MT1039	Rv1010	PROBABLE DIMETHYLADENOSINE TRANSFERASE KSGA(KASUGAMYCIN DIMETHYLTRANSFERASE)
MT1056	Rv1027c	PROBABLE TRANSCRIPTIONAL REGULATORY PROTEIN KDPE
MT1087	Rv1057	CONSERVED HYPOTHETICAL PROTEIN
MT1103	Rv1073	CONSERVED HYPOTHETICAL PROTEIN
MT1106	Rv1076	POSSIBLE LIPASE LIPU
MT1108	Rv1077	PROBABLE CYSTATHIONINE BETA-SYNTHASE CBS (SERINE SULFHYDRASE)
MT1133	Rv1101c	CONSERVED MEMBRANE PROTEIN

MT1136		
MT1153	Rv1121	PROBABLE GLUCOSE-6-PHOSPHATE 1-DEHYDROGENASE ZWF1 (G6PD)
MT1187	Rv1153c	PROBABLE O-METHYLTRANSFERASE OMT
MT1188	Rv1154c	HYPOTHETICAL PROTEIN
MT1190		
MT1213	Rv1176c	CONSERVED HYPOTHETICAL PROTEIN
MT1218	Rv1181	PROBABLE POLYKETIDE BETA-KETOACYL SYNTHASE PKS4
MT1222	Rv1185c	PROBABLE FATTY-ACID--COA LIGASE FADD21
MT1234	Rv1196	PPE FAMILY PROTEIN
MT1238	Rv1200	PROBABLE CONSERVED INTEGRAL MEMBRANE TRANSPORT PROTEIN
MT1247	Rv1209	CONSERVED HYPOTHETICAL PROTEIN
MT1254	Rv1216c	PROBABLE CONSERVED INTEGRAL MEMBRANE PROTEIN
MT1255	Rv1217c	PROBABLE TETRONASIN-TRANSPORT INTEGRAL MEMBRANE PROTEIN ABC TRANSPORTER
MT1258	Rv1220c	PROBABLE METHYLTRANSFERASE
MT1272	Rv1234	PROBABLE TRANSMEMBRANE PROTEIN
MT1281	Rv1244	PROBABLE LIPOPROTEIN LPQZ
MT1298	Rv1260	PROBABLE OXIDOREDUCTASE
MT1301	Rv1263	PROBABLE AMIDASE AMIB2 (AMINOHYDROLASE)
MT1302-1	Rv1264	CONSERVED HYPOTHETICAL PROTEIN
MT1302-2	Rv1264	ADENYLYL CYCLASE (ATP PYROPHOSPHATE-LYASE) (ADENYLATE CYCLASE)
MT1311	Rv1273c	PROBABLE DRUGS-TRANSPORT TRANSMEMBRANE ATP-BINDING PROTEIN ABC TRANSPORTER
MT1326	Rv1288	CONSERVED HYPOTHETICAL PROTEIN
MT1354	Rv1314c	CONSERVED HYPOTHETICAL PROTEIN
MT1363.1	Rv1322	CONSERVED HYPOTHETICAL PROTEIN
MT1370	Rv1328	PROBABLE GLYCOGEN PHOSPHORYLASE GLGP
MT1371	Rv1329c	PROBABLE ATP-DEPENDENT HELICASE DING
MT1390	Rv1348	PROBABLE DRUGS-TRANSPORT TRANSMEMBRANE ATP-BINDING PROTEIN ABC TRANSPORTER
MT1406	Rv1361c	PPE FAMILY PROTEIN
MT1416	Rv1371	PROBABLE CONSERVED MEMBRANE PROTEIN
MT1431	Rv1387	PPE FAMILY PROTEIN
MT1438	Rv1393c	PROBABLE MONOXYGENASE
MT1477	Rv1433	POSSIBLE CONSERVED EXPORTED PROTEIN
MT1495	Rv1448c	PROBABLE TRANSALDOLASE TAL
MT1534-1	Rv1489	CONSERVED HYPOTHETICAL PROTEIN
MT1534-2	Rv1489	CONSERVED HYPOTHETICAL PROTEIN
MT1537		
MT1543	Rv1496	POSSIBLE TRANSPORT SYSTEM KINASE
MT1550	Rv1501	CONSERVED HYPOTHETICAL PROTEIN
MT1553	Rv1505c	CONSERVED HYPOTHETICAL PROTEIN
MT1558		PROBABLE MEMBRANE BOUND POLYKETIDE SYNTHASE PKS6
MT1560		
MT1562	Rv1512	PROBABLE NUCLEOTIDE-SUGAR EPIMERASE EPIA
MT1568	Rv1518	CONSERVED HYPOTHETICAL PROTEIN

MT1577	Rv1526c	PROBABLE GLYCOSYLTRANSFERASE
MT1592	Rv1540	CONSERVED HYPOTHETICAL PROTEIN MEMBER OF YABO/YCEC/YFII FAMILY
MT1593	Rv1541c	POSSIBLE LIPOPROTEIN LPRI
MT1605	Rv1554	PROBABLE FUMARATE REDUCTASE
MT1622.1		
MT1626	Rv1591	PROBABLE TRANSMEMBRANE PROTEIN
MT1650.1		
MT1659	Rv1623c	PROBABLE INTEGRAL MEMBRANE CYTOCHROME D UBIQUINOL OXIDASE
MT1661	Rv1625c	MEMBRANE-ANCHORED ADENYLYL CYCLASE CYA
MT1675	Rv1638	PROBABLE EXCINUCLEASE ABC (SUBUNIT A - DNA-BINDING ATPASE) UVRA
MT1686	Rv1648	PROBABLE TRANSMEMBRANE PROTEIN
MT1708	Rv1670	CONSERVED HYPOTHETICAL PROTEIN
MT1710	Rv1672c	PROBABLE CONSERVED INTEGRAL MEMBRANE TRANSPORT PROTEIN
MT1711	Rv1673c	CONSERVED HYPOTHETICAL PROTEIN
MT1712	Rv1674c	PROBABLE TRANSCRIPTIONAL REGULATORY PROTEIN
MT1727.1	Rv1688	POSSIBLE 3-METHYLADENINE DNA GLYCOSYLASE MPG
MT1741	Rv1702c	CONSERVED HYPOTHETICAL PROTEIN
MT1743	Rv1703c	PROBABLE CATECHOL-O-METHYLTRANSFERASE
MT1744	Rv1704c	PROBABLE D-SERINE/ALANINE/GLYCINE TRANSPORTER PROTEIN CYCA
MT1751	Rv1710	CONSERVED HYPOTHETICAL PROTEIN
MT1767	Rv1726	PROBABLE OXIDOREDUCTASE
MT1778	Rv1736c	PROBABLE NITRATE REDUCTASE NARX
MT1783	Rv1741	CONSERVED HYPOTHETICAL PROTEIN
MT1796	Rv1753c	PPE FAMILY PROTEIN
MT1814.2		
MT1820	Rv1769	CONSERVED HYPOTHETICAL PROTEIN
MT1820.1	Rv1770	CONSERVED HYPOTHETICAL PROTEIN
MT1848	Rv1799	PROBABLE LIPOPROTEIN LPPT
MT1854	Rv1804c	CONSERVED HYPOTHETICAL PROTEIN
MT1858	Rv1810	CONSERVED HYPOTHETICAL PROTEIN
MT1859	Rv1811	POSSIBLE MG2+ TRANSPORT P-TYPE ATPASE C MGTC
MT1866	Rv1818c	PE-PGRS FAMILY PROTEIN
MT1884	Rv1836c	CONSERVED HYPOTHETICAL PROTEIN
MT1918	Rv1869c	PUTATIVE PROTEIN USFY
MT1928	Rv1879	CONSERVED HYPOTHETICAL PROTEIN
MT1933	Rv1885c	CONSERVED HYPOTHETICAL PROTEIN
MT1935	Rv1887	HYPOTHETICAL PROTEIN
MT1937	Rv1889c	CONSERVED HYPOTHETICAL PROTEIN
MT1945		CONSERVED HYPOTHETICAL PROTEIN
MT1959	Rv1908c	CATALASE-PEROXIDASE-PEROXYNITRITASE T KATG
MT1965	Rv1914c	CONSERVED HYPOTHETICAL PROTEIN
MT1970	Rv1919c	CONSERVED HYPOTHETICAL PROTEIN
MT1980		
MT1982	Rv1932	PROBABLE THIOL PEROXIDASE TPX
MT1988	Rv1938	PROBABLE EPOXIDE HYDROLASE EPHB (EPOXIDE HYDRATASE)

MT2017	Rv1965	CONSERVED HYPOTHETICAL INTEGRAL MEMBRANE PROTEIN YRBE3B
MT2029	Rv1977	POSSIBLE HEMOLYSIN-LIKE PROTEIN
MT2030	Rv1978	CONSERVED HYPOTHETICAL PROTEIN
MT2040	Rv1986	PROBABLE CONSERVED INTEGRAL MEMBRANE PROTEIN
MT2061	Rv2005c	CONSERVED HYPOTHETICAL PROTEIN
MT2073.3	Rv2309A	HYPOTHETICAL PROTEIN
MT2081		
MT2082-1	Rv2024c	CONSERVED HYPOTHETICAL PROTEIN
MT2082-2	Rv2024c	CONSERVED HYPOTHETICAL PROTEIN
MT2096	Rv2036	CONSERVED HYPOTHETICAL PROTEIN
MT2101	Rv2041c	PROBABLE SUGAR-BINDING LIPOPROTEIN
MT2104	Rv2044c	CONSERVED HYPOTHETICAL PROTEIN
MT2106	Rv2046	PROBABLE LIPOPROTEIN LPPI
MT2108	Rv2048c	PROBABLE POLYKETIDE SYNTHASE PKS12
MT2112	Rv2052c	CONSERVED HYPOTHETICAL PROTEIN
MT2120	Rv2061c	CONSERVED HYPOTHETICAL PROTEIN
MT2121	Rv2062c	PROBABLE COBALAMIN BIOSYNTHESIS PROTEIN COBN
MT2131	Rv2071c	PROBABLE PRECORRIN-4 C11-METHYLTRANSFERASE COBM
MT2132	Rv2072c	PROBABLE PRECORRIN-6Y METHYLTRANSFERASE COBL
MT2137	Rv2077c	PE FAMILY PROTEIN
MT2149	Rv2088	PROBABLE TRANSMEMBRANE SERINE/THREONINE-PROTEIN KINASE J
MT2152	Rv2091c	PROBABLE MEMBRANE PROTEIN
MT2160	Rv2100	CONSERVED HYPOTHETICAL PROTEIN
MT2173	Rv2113	PROBABLE INTEGRAL MEMBRANE PROTEIN
MT2178-1	Rv2118c	POSSIBLE RNA METHYLTRANSFERASE
MT2178-2	Rv2118c	POSSIBLE RNA METHYLTRANSFERASE
MT2191	Rv2133c	CONSERVED HYPOTHETICAL PROTEIN
MT2208	Rv2149c	CONSERVED HYPOTHETICAL PROTEIN YFIH
MT2236	Rv2181	PROBABLE CONSERVED INTEGRAL MEMBRANE PROTEIN
MT2261	Rv2205c	CONSERVED HYPOTHETICAL PROTEIN
MT2273	Rv2216	CONSERVED HYPOTHETICAL PROTEIN
MT2285.2		
MT2291		
MT2316		
MT2319	Rv2258c	POSSIBLE TRANSCRIPTIONAL REGULATORY PROTEIN
MT2347	Rv2290	PROBABLE CONSERVED LIPOPROTEIN LPPO
MT2359	Rv2302	CONSERVED HYPOTHETICAL PROTEIN
MT2375		
MT2394	Rv2332	PROBABLE [NAD] DEPENDENT MALATE OXIDOREDUCTASE MEZ
MT2395-1	Rv2333c	PROBABLE CONSERVED INTEGRAL MEMBRANE TRANSPORT PROTEIN
MT2395-2	Rv2333c	PROBABLE CONSERVED INTEGRAL MEMBRANE TRANSPORT PROTEIN
MT2405		
MT2432	Rv2363	PROBABLE AMIDASE AMIA2 (AMINOHYDROLASE)
MT2439	Rv2370c	CONSERVED HYPOTHETICAL PROTEIN
MT2444	Rv2375	CONSERVED HYPOTHETICAL PROTEIN
MT2453	Rv2385	PUTATIVE ACETYL HYDROLASE MBTJ
MT2456-1	Rv2387	CONSERVED HYPOTHETICAL PROTEIN

MT2456-2	Rv2387	CONSERVED HYPOTHETICAL PROTEIN
MT2465	Rv2395	PROBABLE CONSERVED INTEGRAL MEMBRANE PROTEIN
MT2509	Rv2435c	PROBABLE CYCLASE (ADENYLYL- OR GUANYLYL-)(ADENYLATE- OR GUANYLATE-)
MT2530	Rv2455c	PROBABLE OXIDOREDUCTASE (ALPHA SUBUNIT)
MT2534	Rv2459	PROBABLE CONSERVED INTEGRAL MEMBRANE TRANSPORT PROTEIN
MT2554.1		
MT2554.1A		
MT2559	Rv2485c	PROBABLE CARBOXYLESTERASE LIPQ
MT2577	Rv2502c	PROBABLE CONSERVED ATP-BINDING PROTEIN ABC TRANSPORTER
MT2601.1		
MT2607	Rv2531c	PROBABLE REDUCTASE
MT2618	Rv2543	PROBABLE CONSERVED LIPOPROTEIN LPPA
MT2634	Rv2557	CONSERVED HYPOTHETICAL PROTEIN
MT2636	Rv2559c	CONSERVED HYPOTHETICAL ALANINE LEUCINE VALINE RICH PROTEIN
MT2638	Rv2562	CONSERVED HYPOTHETICAL PROTEIN
MT2656	Rv2579	PROBABLE EXCINUCLEASE ABC (SUBUNIT B - HELICASE) UVRB
MT2665	Rv2588c	PROBABLE CONSERVED MEMBRANE PROTEIN SECRETION FACTOR YAJC
MT2667	Rv2590	PROBABLE FATTY-ACID-COA LIGASE FADD9
MT2673	Rv2597	PROBABLE MEMBRANE PROTEIN
MT2683-1	Rv2608	PPE FAMILY PROTEIN
MT2683-2	Rv2608	PPE FAMILY PROTEIN
MT2701	Rv2626c	CONSERVED HYPOTHETICAL PROTEIN
MT2707	Rv2631	CONSERVED HYPOTHETICAL PROTEIN
MT2712	Rv2634c	UDP-GLUCOSE 4-EPIMERASE GALE1 (GALACTOWALDENASE)
MT2719	Rv2641	CADMIUM INDUCIBLE PROTEIN CADI
MT2734	Rv2657c	PROBABLE MOLYBDENUM COFACTOR BIOSYNTHESIS PROTEIN C MOAC1
MT2760	Rv2686c	PROBABLE ANTIBIOTIC-TRANSPORT INTEGRAL MEMBRANE
MT2764	Rv2690c	PROBABLE CONSERVED INTEGRAL MEMBRANE
MT2768	Rv2694c	CONSERVED HYPOTHETICAL PROTEIN
MT2795	Rv2723	PROBABLE CONSERVED INTEGRAL MEMBRANE PROTEIN
MT2797	Rv2725c	PROBABLE GTP-BINDING PROTEIN HFLX
MT2804	Rv2735c	CONSERVED HYPOTHETICAL PROTEIN
MT2806	Rv2737c	RECA PROTEIN (RECOMBINASE A) [CONTAINS: ENDONUCLEASE PI-MTUI (MTU RECA INTEIN)].
MT2810		CONSERVED 13E12 REPEAT FAMILY PROTEIN
MT2812	Rv2741	PE-PGRS FAMILY PROTEIN
MT2819	Rv2748c	POSSIBLE CELL DIVISION TRANSMEMBRANE PROTEIN FTSK
MT2820	Rv2750	PROBABLE DEHYDROGENASE
MT2835	Rv2765	PROBABLE ALANINE RICH HYDROLASE
MT2840-1	Rv2770c	PPE FAMILY PROTEIN
MT2850-2	Rv2780	SECRETED L-ALANINE DEHYDROGENASE ALD (40 KDA ANTIGEN) (TB43)
MT2858	Rv2788	PROBABLE TRANSCRIPTIONAL REPRESSOR SIRR
MT2865	Rv2796c	PROBABLE CONSERVED LIPOPROTEIN LPPV
MT2867.1	Rv2799	PROBABLE MEMBRANE PROTEIN
MT2876	Rv2809	HYPOTHETICAL PROTEIN
MT2886	Rv2819c	HYPOTHETICAL PROTEIN

MT2892	Rv2825c	CONSERVED HYPOTHETICAL PROTEIN
MT2916	Rv2850c	POSSIBLE MAGNESIUM CHELATASE
MT2934		
MT2945	Rv2877c	PROBABLE CONSERVED INTEGRAL MEMBRANE PROTEIN
MT2947	Rv2879c	CONSERVED HYPOTHETICAL PROTEIN
MT2962	Rv2894c	PROBABLE INTEGRASE/RECOMBINASE XERC
MT2963	Rv2895c	POSSIBLE MYCOBACTIN UTILIZATION PROTEIN VIUB
MT2979	Rv2911	PROBABLE D-ALANYL-D-ALANINE CARBOXYPEPTIDASE DACB2
MT2985	Rv2917	CONSERVED HYPOTHETICAL ALANINE AND ARGININE RICH PROTEIN
MT2988	Rv2920c	PROBABLE AMMONIUM-TRANSPORT INTEGRAL MEMBRANE PROTEIN AMT
MT2994	Rv2924c	PROBABLE FORMAMIDOPYRIMIDINE-DNA GLYCOSYLASE FPG (FAPY-DNA GLYCOSYLASE)
MT3006	Rv2936	PROBABLE DAUNORUBICIN-DIM-TRANSPORT ATP-BINDING PROTEIN ABC TRANSPORTER DRRA
MT3015	Rv2943	PROBABLE TRANSPOSASE FOR INSERTION SEQUENCE ELEMENT IS1533
MT3034	Rv2958c	POSSIBLE GLYCOSYL TRANSFERASE
MT3037		
MT3045	Rv2967c	PROBABLE PYRUVATE CARBOXYLASE PCA (PYRUVIC CARBOXYLASE)
MT3051	Rv2973c	PROBABLE ATP-DEPENDENT DNA HELICASE RECG
MT3060	Rv2982c	PROBABLE GLYCEROL-3-PHOSPHATE DEHYDROGENASE
MT3085	Rv3005c	CONSERVED HYPOTHETICAL PROTEIN
MT3093	Rv3013	CONSERVED HYPOTHETICAL PROTEIN
MT3134	Rv3049c	PROBABLE MONOOXYGENASE
MT3143	Rv3057c	PROBABLE SHORT CHAIN ALCOHOL DEHYDROGENASE/REDUCTASE
MT3149	Rv3063	PROBABLE CARBON STARVATION PROTEIN A HOMOLOG CSTA
MT3159	Rv3074	CONSERVED HYPOTHETICAL PROTEIN
MT3170	Rv3085	PROBABLE SHORT-CHAIN TYPE DEHYDROGENASE/REDUCTASE
MT3175	Rv3090	HYPOTHETICAL ALANINE AND VALINE RICH PROTEIN
MT3178	Rv3094c	CONSERVED HYPOTHETICAL PROTEIN
MT3179	Rv3095	HYPOTHETICAL TRANSCRIPTIONAL REGULATORY PROTEIN
MT3203	Rv3121	PROBABLE CYTOCHROME P450 141 CYP141
MT3208	Rv3124	PROBABLE TRANSCRIPTIONAL REGULATORY PROTEIN
MT3216	Rv3130c	CONSERVED HYPOTHETICAL PROTEIN
MT3218	Rv3132c	CONSERVED HYPOTHETICAL PROTEIN
MT3231	Rv3144c	PPE-FAMILY PROTEIN
MT3247	Rv3159c	PPE FAMILY PROTEIN
MT3249	Rv3160c	POSSIBLE TRANSCRIPTIONAL REGULATORY PROTEIN (PROBABLY TETR-FAMILY)
MT3253	Rv3164c	PROBABLE METHANOL DEHYDROGENASE TRANSCRIPTIONAL REGULATORY PROTEIN MOXR3
MT3264	Rv3175	POSSIBLE AMIDASE (AMINOHYDROLASE)
MT3280		CONSERVED HYPOTHETICAL
MT3285	Rv3193c	PROBABLE CONSERVED TRANSMEMBRANE PROTEIN
MT3295	Rv3201c	PROBABLE ATP-DEPENDENT DNA HELICASE
MT3297		
MT3302		
MT3307	Rv3211	PROBABLE ATP-DEPENDENT RNA HELICASE RHLE

MT3310	Rv3214	POSSIBLE PHOSPHOGLYCERATE MUTASE GPM2 (PHOSPHOGLYCEROMUTASE)
MT3321	Rv3224	POSSIBLE IRON-REGULATED SHORT-CHAIN DEHYDROGENASE/REDUCTASE
MT3335	Rv3238c	PROBABLE CONSERVED INTEGRAL MEMBRANE PROTEIN
MT3367	Rv3267	CONSERVED HYPOTHETICAL PROTEIN (CPSA-RELATED PROTEIN)
MT3387	Rv3288c	PUTATIVE PROTEIN USFY
MT3400	Rv3301c	PROBABLE PHOSPHATE-TRANSPORT SYSTEM TRANSCRIPTIONAL REGULATORY PROTEIN PHOU HOMOLOG 1 PHOY1
MT3429		
MT3430	Rv3327	PROBABLE TRANSPOSASE FUSION PROTEIN
MT3431	Rv3328c	PROBABLE ALTERNATIVE RNA POLYMERASE SIGMA FACTOR (FRAGMENT) SIGJ
MT3461	Rv3353c	CONSERVED HYPOTHETICAL PROTEIN
MT3492	Rv3384c	CONSERVED HYPOTHETICAL PROTEIN
MT3511	Rv3403c	HYPOTHETICAL PROTEIN
MT3516	Rv3408	CONSERVED HYPOTHETICAL PROTEIN
MT3518	Rv3410c	PROBABLE INOSINE-5'-MONOPHOSPHATE DEHYDROGENASE GUAB3
MT3534	Rv3430c	POSSIBLE TRANSPOSASE
MT3536		
MT3561	Rv3454	PROBABLE CONSERVED INTEGRAL MEMBRANE PROTEIN
MT3573.3	Rv1582c	PROBABLE PHIRV1 PHAGE PROTEIN
MT3585	Rv3481c	PROBABLE INTEGRAL MEMBRANE PROTEIN
MT3590	Rv3486	CONSERVED HYPOTHETICAL PROTEIN
MT3598	Rv3494c	MCE-FAMILY PROTEIN MCE4F
MT3599	Rv3495c	POSSIBLE MCE-FAMILY LIPOPROTEIN LPRN (MCE-FAMILY LIPOPROTEIN MCE4E)
MT3604	Rv3500c	CONSERVED HYPOTHETICAL INTEGRAL MEMBRANE PROTEIN YRBE4B
MT3613-1		
MT3613-2		
MT3636		
MT3653	Rv3549c	PROBABLE SHORT-CHAIN TYPE DEHYDROGENASE/REDUCTASE
MT3656	Rv3552	POSSIBLE COA-TRANSFERASE (BETA SUBUNIT)
MT3661	Rv3557c	PROBABLE TRANSCRIPTIONAL REGULATORY PROTEIN (PROBABLY TETR-FAMILY)
MT3753	Rv3651	CONSERVED HYPOTHETICAL PROTEIN
MT3773	Rv3672c	CONSERVED HYPOTHETICAL PROTEIN
MT3785	Rv3683	CONSERVED HYPOTHETICAL PROTEIN
MT3807	Rv3704c	GLUTAMATE--CYSTEINE LIGASE GSHA
MT3814	Rv3711c	PROBABLE DNA POLYMERASE III (EPSILON SUBUNIT) DNAQ
MT3826	Rv3723	PROBABLE CONSERVED TRANSMEMBRANE PROTEIN
MT3828	Rv3725	POSSIBLE OXIDOREDUCTASE
MT3830	Rv3727	POSSIBLE OXIDOREDUCTASE
MT3849	Rv3741c	POSSIBLE OXIDOREDUCTASE
MT3862	Rv3755c	CONSERVED HYPOTHETICAL PROTEIN
MT3877	Rv3770c	HYPOTHETICAL PROTEIN
MT3884	Rv3775	PROBABLE LIPASE LIPE

MT3888	Rv3779	PROBABLE CONSERVED TRANSMEMBRANE PROTEIN ALANINE AND LEUCINE RICH
MT3895	Rv3787c	CONSERVED HYPOTHETICAL PROTEIN
MT3900	Rv3793	INTEGRAL MEMBRANE INDOLYLACETYLINOSITOL ARABINOSYLTRANSFERASE EMBC
MT3904	Rv3797	PROBABLE ACYL-COA DEHYDROGENASE FADE35
MT3928	Rv3820c	POSSIBLE CONSERVED POLYKETIDE SYNTHASE ASSOCIATED PROTEIN PAPA2
MT3930	Rv3822	
MT3938	Rv3830c	TRANSCRIPTIONAL REGULATORY PROTEIN (PROBABLY TETR-FAMILY)
MT3954.1		
MT3969	Rv3854c	MONOOXYGENASE ETHA
MT3972	Rv3857c	POSSIBLE MEMBRANE PROTEIN
MT3974.1		
MT3980		
MT3982	Rv3869	POSSIBLE CONSERVED MEMBRANE PROTEIN
MT3985	Rv3871	CONSERVED HYPOTHETICAL PROTEIN
MT3996	Rv3881c	CONSERVED HYPOTHETICAL ALANINE AND GLYCINE RICH PROTEIN
MT4006	Rv3891c	POSSIBLE ESAT-6 LIKE PROTEIN ESXD
MT4024	Rv3905c	PUTATIVE ESAT-6 LIKE PROTEIN ESXF
